# “There's more love between us”: The parental experience of attending Mellow Babies, a targeted, early intervention program for parents and their babies

**DOI:** 10.1002/imhj.22029

**Published:** 2022-12-15

**Authors:** Ciera Davidson, Aigli Raouna, Ruaridh Malcolm, Raquib Ibrahim, Angus MacBeth

**Affiliations:** ^1^ Department of Clinical Psychology School of Health in Social Science The University of Edinburgh Edinburgh UK; ^2^ Mellow Parenting Glasgow UK

**Keywords:** early intervention, infant mental health, Mellow Babies, parent and baby, parenting, Bebés Apacibles (Mellow Babies), crianza, intervención temprana, progenitor y bebé, salud mental infantil, Bébés d'humeur joyeuse, Parentage, Intervention précoce, Parent et bébé, Santé mentale du nourrisson, Mellow Babies, Elternschaft, Frühförderung, Eltern und Baby, psychische Gesundheit von Kleinkindern, メロウ　ベビー、子育て、早期介入、親-乳児、乳幼児のメンタルヘルス, 关键词:“成熟宝宝”, 育儿, 早期干预, 父母和婴儿, 婴儿心理健康, **الكلمات المفتاحية**: الأطفال اليانعون ، التربية الوالدية ، التدخل المبكر ، الوالدين والطفل ، الصحة النفسية للرضع

## Abstract

It is recognized that parenthood in the context of psychosocial adversity can have negative implications for infant development. Parenting programs are the first line of intervention to improve outcomes for families; however, evidence for the effectiveness of group‐based, targeted early interventions is still scarce. Preliminary findings indicate Mellow Babies (MB) as a promising group‐based parenting program for families at risk for parenting difficulties. Using thematic analysis, we aimed to understand: (i) the aspects of the intervention that enabled parents to complete the program and (ii) the relational and behavioral changes perceived as valuable for parents and their babies post‐intervention. In total, 68 parents residing in the United Kingdom were interviewed after completing MB (49 mothers and 19 fathers; 88% self‐identified as British). Three themes and six subthemes were generated from the data. Parents identified several intervention components as beneficial, including the facilitators' interpersonal skills and multi‐dimensional, group‐based approach. Participant reflections highlighted three underlying mechanisms that enabled positive change: (i) the sense of community cultivated within the group, (ii) the process of formulating and re‐conceptualizing one's difficulties, and (iii) the opportunity to reshape interpersonal interactions. Findings are discussed within the context of perinatal and infant mental health.

## Resumen

Se reconoce que la paternidad/maternidad dentro del contexto de la adversidad sicosocial puede tener implicaciones negativas para el desarrollo del infante. Los programas sobre la crianza son la línea frontal de intervención para mejorar los resultados para las familias, sin embargo, la evidencia para la eficacia de las intervenciones tempranamente identificadas, con base en el grupo, es escasa. Los resultados preliminares indican que Bebés Apacibles (Mellow Babies / MB) es un prometedor programa de crianza con base en el grupo para familias bajo riesgo de dificultades en la crianza. Haciendo uso de análisis temáticos, nos propusimos comprender: i) los aspectos de la intervención que les permite a los progenitores completar el programa y ii) los cambios relacionales y de comportamiento que se percibieron como valiosos para los progenitores y sus bebés con posterioridad a la intervención. En total, se entrevistaron 68 progenitores residentes del Reino Unido después de completar MB (49 mamás y 19 papás; 88% autoidentificados como británicos). De la información surgieron tres temas y seis subtemas. Los progenitores identificaron como beneficiosos varios componentes de la intervención, incluyendo las habilidades interpersonales de quienes facilitaban la participación y el acercamiento multidimensional con base en el grupo. Las reflexiones de los participantes subrayaron tres mecanismos subyacentes que permitieron el cambio positivo: i) el sentido de comunidad cultivado dentro del grupo; ii) el proceso de formular y reconceptualizar las propias dificultades; y iii) la oportunidad de volver a darle forma a las interacciones interpersonales. Los resultados se discuten dentro del contexto de la salud mental perinatal y del infante.

## Résumé

On reconnaît généralement que la parentalité dans le contexte d'adversité psychosociale peut avoir des implications négatives pour le développement du bébé. Les programmes de parentage sont la première ligne d'intervention afin d'améliorer les résultats pour les familles. Cependant les preuves de l'efficacité d'interventions précoces ciblées et basées sur un groupe sont encore rares. Des résultats préliminaires indiquent que Bébés d'humeur joyeuse, Mellow Babies (MB) est un programme de parentage basé sur un groupe prometteur pour les familles à risque de difficultés de parentage. En utilisant une analyse thématique nous nous sommes donné pour but de comprendre: i) les aspects de l'intervention qui permettent aux parents de finir le programme et ii) les changements relationnels et comportementaux qui sont perçus comme étant précieux pour les parents et leurs bébé après l'intervention. Au total 68 parents résidant au Royaume Uni ont été interviewés après avoir terminé le programme MB (49 mères et 19 pères; 88% s’étant auto‐identifiés comme britanniques). Trois thèmes et six sous‐thèmes ont émergé des données. Les parents ont identifié plusieurs composantes de l'intervention comme étant bénéfiques, y compris les compétences interpersonnelles du facilitateur ou de la facilitatrice et son approche multi‐dimensionnelle, basée sur un groupe. Les réflexions des participants ont mis en lumière trois mécanismes sous‐jacents qui permettait un changement positif: i) le sens de communauté cultivé au sein du groupe; ii) les processus de formulation et de ré‐conceptualisation de ses propres difficultés; et iii) l'opportunité de refaçonner les interactions interpersonnelles. Les résultats sont discutés dans le contexte de la santé mentale du nourrisson et périnatale.

## Zusammenfassung

“Da ist mehr Liebe zwischen uns”: Erfahrungen von Eltern mit der Teilnahme an „Mellow Babys“, einem gezielten Frühförderprogramm für Eltern und ihre Babys

Es ist bekannt, dass Elternschaft im Kontext von psychosozialen Widrigkeiten negative Auswirkungen auf die Entwicklung von Säuglingen haben kann. Elternprogramme sind die erste Anknüpfungsstelle, um Outcomes für Familien zu verbessern, doch gibt es bisher nur wenig Wirksamkeitsbelege für gruppenbasierte, gezielte Frühinterventionen. Vorläufige Ergebnisse deuten darauf hin, dass „Mellow Babies“ (MB) ein vielversprechendes gruppenbasiertes Elternprogramm für Familien mit einem Risiko für Erziehungsschwierigkeiten ist. Mithilfe einer thematischen Analyse wollten wir zwei Dinge besser verstehen: i) Welche Aspekte der Intervention versetzen die Eltern dazu in der Lage, das Programm abzuschließen, und ii) Welche Beziehungs‐ und Verhaltensänderungen werden nach der Intervention für die Eltern und ihre Babys als wertvoll empfunden wurden. Insgesamt wurden 68 Eltern mit Wohnsitz in Großbritannien nach dem Abschluss von MB interviewt (49 Mütter und 19 Väter; 88 % gaben an, Briten zu sein). Aus den Daten ergaben sich drei Themen und sechs Unterthemen. Die Eltern bezeichneten mehrere Komponenten der Intervention als hilfreich, darunter die zwischenmenschlichen Fähigkeiten der Moderator:in und den multidimensionalen, gruppenbasierten Ansatz. In den Reflexionen der Teilnehmenden wurden drei zugrundeliegende Mechanismen hervorgehoben, die positive Veränderungen ermöglichten: i) Das in der Gruppe kultivierte Gemeinschaftsgefühl, ii) der Prozess der Formulierung und Neukonzeption eigener Schwierigkeiten; und iii) die Möglichkeit, zwischenmenschliche Interaktionen neu zu gestalten. Die Ergebnisse werden im Kontext der perinatalen und infantilen psychischen Gesundheit diskutiert.

## 抄録

“私たちの愛が深まった”: 親と赤ちゃんのための焦点化された早期介入プログラム「Mellow Babiesメロウ ベイビーズ」に参加した親の体験

心理社会的逆境という文脈の中での子育ては、乳幼児の発達に悪影響を及ぼすと認識されている。子育て支援プログラムは、家族の未来を改善するための介入手段の第一選択肢であるが、集団を基にしてターゲットを絞った早期介入の有効性を示す根拠はまだ乏しい。予備的知見では、メロウベイビーズ (MB) は、子育て困難のリスクを抱える家族向けの集団を基にした子育てプログラムとして有望であることが示された。我々は、テーマ分析を用いて、i) 親をプログラム修了に導いた、介入の側面、ii) 介入後に親とその赤ちゃんにとって価値があると認識された関係性と行動の変化、を理解することを目的とした。英国在住の68名の保護者 (母親49名、父親19名、88％が英国人と自認) に、MB終了後にインタビューを行った。その結果、3つのテーマと6つのサブテーマがデータから浮かび上がった。親は、ファシリテーターの対人関係スキルや多次元的な集団を基にしたアプローチなど、いくつかの介入要素を有益であると認識した。参加者の考察では、肯定的な変化を可能にする3つの基本的なメカニズムが強調された。i) グループ内で培われた共同体感覚、ii) 自分の困難を定式化し再認識するプロセス、iii) 対人相互交流を再形成する機会である。これらの知見は、周産期・乳幼児期のメンタルヘルスという文脈にそって議論されている。

## 摘要

人们认识到, 在社会心理逆境中为人父母会对婴儿的发育产生消极影响。育儿计划是改善家庭发展结果的第一道干预线, 然而, 基于群体的、有针对性的早期干预措施有效性的证据仍然很少。初步研究结果表明, “成熟宝宝” (MB) 是一种很有前途的基于群体的育儿计划, 适用于有育儿困难风险的家庭。通过主题分析, 我们旨在了解:1) 使父母能够完成计划的干预措施;2) 在干预后被认为对父母及其婴儿有价值的关系和行为变化。总共有68位居住在英国的父母在完成MB后接受了采访 (49位母亲和19位父亲;88%的人自称是英国人) 。数据中归纳出三个主题和六个子主题。父母们确定了几个有益的干预因素, 包括辅导者的人际交往技能及其多维度、基于小组的方法。参与者的反映强调了促成积极变化的三个潜在机制:1) 群体内培养的社区意识;2) 阐述和重新概念化困难的过程;3) 重塑人际交往的机会。研究结果在围产期和婴儿心理健康的背景下进行了讨论。

## ملخص

من المعروف أن الرعاية الوالدية في ظل الشدائد النفسية والاجتماعية يمكن أن يكون لها آثار سلبية على نمو الرضع. وتعتبر برامج التربية الوالدية هي خط التدخل الأول لتحسين النتائج للعائلات ، ومع ذلك ، لا تزال الأدلة على فعالية التدخلات المبكرة المستهدفة والمرتكزة على المجموعة نادرة. تشير النتائج الأولية إلى أن برنامج الأطفال اليانعين (Mellow Babies, MB) يعد برنامجًا واعدًا قائماً على المجموعة يختص بصعوبات التربية الوالدية وموجه للعائلات المعرضة للمخاطرة. باستخدام التحليل المواضيعي ، كنا نهدف إلى فهم 1) جوانب التدخل التي مكنت الآباء من إكمال البرنامج و 2) التغييرات العلائقية والسلوكية التي كانت ذات قيمة في نظر الوالدين وأطفالهم بعد التدخل. في المجموع ، تمت مقابلة 68 من الآباء والأمهات المقيمين في المملكة المتحدة بعد إكمال البرنامج (88 ٪ عرفوا بأنفسهم على أنهم بريطانيون). ظهرت ثلاثة مواضيع رئيسية وستة مواضيع فرعية من البيانات. حدد الآباء العديد من مكونات التدخل على أنها مفيدة ، بما في ذلك مهارات التواصل لمنسق البرنامج ونهج التدخل متعدد الأبعاد القائم على المجموعة. سلطت استجابات المشاركين الضوء على ثلاث آليات أساسية ساعدت على إحداث تغيير إيجابي: 1) الشعور بالمجتمع الذي ينمو داخل المجموعة ؛ 2) عملية صياغة وإعادة تصور الصعوبات الشخصية ؛ و 3) فرصة إعادة تشكيل التفاعلات الشخصية. يتم مناقشة النتائج في سياق الصحة النفسية في الفترة المحيطة بالولادة والرضع.

## 1 INTRODUCTION

Growing international research evidence and public health initiatives highlight infancy, specifically the first 2 years of life, as a sensitive and critical period from which future behavioral, cognitive, emotional, and social development can be shaped (Nelson et al., 2019; Williams, 2019). Exposure to contextual and psychosocial adversities during early childhood, such as poverty (Playford et al., 2017), domestic violence (Margolin et al., 2010), and poor parental mental health (Reupert & Mayberry, 2016), can impact the socio‐emotional development of babies and the quality of their relationship with primary caregivers (Cadar et al., 2018; McDonald et al., 2016; Speidel et al., 2020). The cumulative effect of low economic and psychosocial resources (including but not limited to unemployment, low parental education, single parenthood, and social isolation) can increase family stress which may limit the practical and emotional support parents can provide their children (January et al., 2017; Stein et al., 2014). Nevertheless, emerging evidence suggests that promoting sensitive and nurturing relationships within the early years can mitigate the long‐term impact of psychosocial adversity (McElroy & Hevey, 2014; Parkes et al., 2016; Potharst et al., 2018). Therefore, the effective implementation of early intervention programs targeting parents that experience one or more of the above risk indicators (henceforth; at‐risk families) could have significant short and long‐term public health benefits.

Existing evidence suggests that parenting interventions can benefit families irrespective of risk status when these are tailored to their needs. For example, a meta‐analysis of 133 different early intervention programs starting either during pregnancy or in the first 6 months postnatally (of which 2/3 targeted at‐risk families) revealed very small to small significant positive effects on parenting behaviors, parental stress and mental health, as well as child developmental outcomes, which did not vary by the risk status of the participants (Pinquart & Teubert, 2010). Corroborating these results, a more recent meta‐analysis evaluating 16 parenting interventions offered to at‐risk caregivers with infants up to 12 months old indicated a small but statistically significant positive effect of the interventions on child behavior as well as moderate effects on the parent‐child relationship and maternal sensitivity (Rayce et al., 2017). These findings demonstrate that parenting interventions initiated during the first year of children's lives, despite earlier reported inconsistent findings (e.g., Sweet & Appelbaum, 2004), have the potential to improve parental wellbeing, child behavior, and the quality of parent‐infant relationships post‐intervention regardless of the level of parental psychosocial adversity. However, as evidenced by the above reviews, the majority of existing early parenting programs targeting at‐risk families are either delivered individually or in couples, leaving a gap in our knowledge about whether group‐based, targeted early interventions can also be effective and if so, what intervention components can mediate positive change.

Current research on the effectiveness of group‐based parenting interventions is mainly available for families with middle‐to‐high socioeconomic status and mother‐child dyads (Barlow et al., 2016; Jones et al., 2016; Wilson et al., 2012). Therefore, the generalizability of group‐based intervention benefits is limited by a high drop‐out rate of at‐risk families (Smokowski et al., 2018; Utting et al., 2007) and a generally low participation of fathers, similar to home‐based interventions (Panter‐Brick et al., 2014; Pinquart & Teubert, 2010). In the UK context, a range of complex, interlinking and multilevel barriers and enablers to accessing and engaging with postnatal services have been identified both from the perspective of mothers (Smith et al., 2019; Webb et al., 2021) and fathers (Darwin et al., 2017; Sicouri et al., 2018). Multiple factors have been reported to constitute barriers, including personal life factors (e.g., symptoms of psychosocial difficulties and limited resources), interpersonal factors (e.g., lack of trusting relationships and open communication with healthcare providers), and program‐specific factors (e.g., gender‐specific content and mode of delivery). Feelings of stigmatization or guilt appear to disproportionately affect at‐risk parents who have to navigate parenthood challenges within the realm of stressful life circumstances, further discouraging them from engaging in parenting interventions due to their fear of being judged (Mytton et al., 2014). Barriers specific to fathers’ involvement in postnatal services draw attention to additional factors, such as perceptions that available interventions are mother‐baby oriented and lack relevance to them, as well as personal and systemic beliefs about gender roles regarding fatherhood and help‐seeking behaviors (Sicouri et al., 2018).

It is well established that tailored parenting interventions delivered within the home environment can engage and support at‐risk families by overcoming several of the practical and logistical barriers mentioned above (Kitzman et al., 2019; Olds et al., 2019). However, their delivery cost is estimated to be approximately six times more than that of group interventions which may limit their scope of provision and availability (Cunningham et al., 1995; Jones et al., 2016; Lee et al., 2012). Alternatively, interventions delivered within community settings may offer additional benefits to the families, dismantling socio‐contextual barriers by reducing social isolation and facilitating community building (Butler et al., 2020; Ruane et al., 2019). Therefore, gaining a better understanding of what implementation components and processes can facilitate the engagement of at‐risk families in early parenting group‐based programs could provide valuable insights for the refinement of group interventions, potentially reaching and benefiting more families in need as a less resource‐intensive alternative to home visiting.

### 1.1 Mellow Babies: A group‐based early parenting intervention

Mellow Babies (MB) is part of the Mellow Parenting (MP) family of parenting programs (https://www.mellowparenting.org/) offering targeted support to parents experiencing psychosocial difficulties with infants up to 18 months old. The intervention has no cost to attendees and includes gender‐specific postnatal programs delivered separately through community‐based services in groups of 5–10 parents (Mellow Mums and Mellow Dads). The groups run for 14 weeks, one full day a week, and employs a range of strategies to reduce barriers related to engagement and attendance by providing transport, meals, free parallel childcare, and free or inexpensive materials for parent‐child activities. Parent‐baby dyads who attend at least 70% of the group sessions (i.e., minimum 10/14 sessions) are considered program completers. Health and social care professionals working with families and young children (e.g., health visitors, midwives, social care workers) can deliver MB following training. Ongoing supervision for practitioners is provided, which is essential for their accreditation. Two to three practitioners facilitate each group, of which at least one must match the gender of the parents in the group.

The intervention adopts a dual approach aiming to strengthen parent‐child relationships while improving parental mental health and confidence in the parental role. In line with attachment‐informed interventions, parental sensitivity and parent‐infant synchronicity are nurtured via the provision of child development psycho‐education and guided positive interaction through strength‐based video feedback (Bakermans‐Kranenburg et al., 2003). MB also incorporates parental mental health strategies (e.g., cognitive‐behavioral strategies to cope with depression and anxiety symptoms), parent‐child relationship components (e.g., joint lunchtime activities to enhance parent‐child interactions), and “homework” (e.g., new skills and baby activities to try out at home). Each session follows the same structure; a morning personal group (focusing on topics such as Trust, Depression, You and Your Body and Self‐Esteem); a lunchtime child activity (e.g., nursery rhymes, music or hand painting which aim to equip parents with practical tools that enable them to interact in a playful and developmentally‐appropriate way with their child, using free or inexpensive materials) and; an afternoon parenting workshop (e.g., discussing parenting with topics like Understanding, Keeping Children Busy, Feelings, and sharing parent videos).

Mellow Parenting (MP) has a growing evidence base demonstrating medium positive effect sizes for parents’ mental health and wellbeing, parenting confidence, child outcomes and perceived parent‐child closeness (Levi et al., 2019; MacBeth et al., 2015; Puckering et al., 2010; Raouna et al., 2021). Completion of MP programs has been associated with reductions in the involvement of child protection services (Raouna et al., 2021) and conduct problems in early childhood (Levi et al., 2019). Preliminary findings suggest that MB can equally benefit families regardless of risk status and gender and retain a higher proportion of at‐risk parent‐infant dyads than other group‐based parenting programs (Raouna et al., 2021). However, given the lack of control groups and the relatively small sample sizes, these findings should be interpreted cautiously. Additionally, although these positive MP outcomes have been supported by small‐scale qualitative studies (Birtwell et al., 2015; Puckering et al., 1996, 2010), there is limited evidence specific to understanding the underlying mechanisms that can elicit change and engagement for parent‐baby dyads participating in the MB program. Preliminary qualitative investigations of MP programs have indicated three components of the interventions as potential mechanisms of change: (i) the psycho‐education on depression and child development, (ii) the acquisition of a social support network (Puckering et al., 2010), and (iii) the opportunity to reflect on past interpersonal life experiences within the group (Birtwell et al., 2015; Buston et al., 2019). However, these are all maternal reflections and are not specific to the MB intervention. Therefore, there is still little knowledge about what factors at‐risk mothers and fathers, who are typically less likely to engage with group‐based parenting interventions, identify as accessible and meaningful in the MB program.

### 1.2 Aims

This is the first larger‐scale study to qualitatively gather information both from mothers and fathers completing the MB program in UK‐wide community settings, providing the opportunity to triangulate what aspects of MB work for parents attending the intervention in different contexts. We aimed to gain a qualitative insight into the parental experience of attending MB and understand what components and processes were perceived as meaningful to parents and encouraged the completion of MB. Specifically, we were interested in uncovering:

1. What did parents perceive as meaningful and engaging in the design and delivery of MB?
2. What did parents identify as valuable interpersonal, relational and behavioral change for them and their infant post‐intervention?

## 2 METHODS

### 2.1 Ethical approval

Ethical approval for this project was granted by the Research Ethics Committee of the School of Health in Social Science, the University of Edinburgh. The study was registered with ISRCTN: Registration number ISRCTN17621046.

### 2.2 Design

This report is part of a mixed‐methods project that employed a pragmatic pre‐post‐intervention design. A pragmatic trial design was used to evaluate the effectiveness of MB in “real‐life” routine practice conditions, achieving maximum external validity and producing results that can be generalized and applied in UK routine practice settings (Patsopoulos, 2011). Here we focus on the qualitative data collected as part of this project via semi‐structured interviews with parents who completed the MB groups. For a detailed overview of the quantitative aspect of this project, refer to Raouna et al. (2021).

### 2.3 Group and participant recruitment

Participants were recruited using opportunistic sampling. All naturally occurring MB groups facilitated by MP trained practitioners in community‐based centers across the UK from February 2017 to September 2018 were invited to participate in the study via emails, posters or personal communication. To ensure program fidelity, at least one of the group practitioners had to have previous experience in supervised MB delivery and MB groups had to implement all elements and values of MB to be eligible for this research project (e.g., at least one gender‐specific practitioner in the group, weekly sessions including strength‐based video feedback, free parallel childcare, parent‐infant joint lunchtime activities, and provision of transportation and meals). Fifteen groups were recruited, of which 10 were delivered in Scotland (five Mellow Mums and five Mellow Dads), three in England (Mellow Mums), and two in Northern Ireland (Mellow Mums). An incentive of £250 towards the cost of a group activity was offered to each participating MB group.

Participating centers facilitated the recruitment of parents via MP's usual referral pathways (referred by a health‐related professional or, less often, self‐referred). Practitioners confirmed eligibility to participate in the MB groups for all referred parents. Parents had to have at least one child under the age of 18 months that could attend at lunchtime and in joint activities each week, and parents had to agree to have a video recorded during a caretaking activity (e.g., during feeding) for the strength‐based video feedback element of the intervention. Although MP does not advise formal screening of the psychosocial status of referred parents, referrals are usually targeted to parents identified as experiencing a mental health issue (commonly depression and/or anxiety), isolation, unemployment, domestic violence, substance use, difficulties in parental role, social work involvement, and involvement in child protection services. For the purposes of this research project, participating parents were categorized as either at‐risk or not at‐risk by the research team. A parent was considered at‐risk if they lived in SIMD decile 1 or 2 (Scottish Index of Multiple Deprivation: http://simd.scot/2016/#/simd2016/BTTTFTT/9/‐4.0000/55.9000; for Scotland‐based families only, SIMD data can be split into deciles with decile 1 representing the 10% most deprived postcodes and decile 10 representing the 10% least deprived postcodes); if they reported experiencing a mental health issue; or if they reported being a single parent and being unemployed.

Group facilitators informed parents about the study in their initial contact and clarified that participation in the study would not affect their involvement in the MB group. Two research team members visited each MB group in person before or during the first session to provide more detailed information. Group members were informed that participation in the study was voluntary and confidential and that only anonymized findings would be disseminated. All parents who opted to participate were entered into a prize draw to win one of six £40 supermarket vouchers. Participants provided written informed consent.

### 2.4 Procedure

Data collection was conducted by three experienced MSc‐graduate researchers employed by MP who were not blind to the study aims (A.R., R.M., R.I.). The researchers collected the data during a 1:1 session with each parent in a familiar, private space within the group service facilities at two time points (T): T1 – pre‐group (baseline demographics and self‐reported questionnaires) and T2 – post‐group (self‐reported questionnaires and semi‐structured interview). During both time points, the researchers adopted a respectful and discussion‐based approach to minimize perceived power imbalances, establish trust and create a non‐judgmental and safe environment for participants. In addition, to enable building a researcher‐parent rapport, an essential element for parents to feel at ease and openly share their experiences, the same researcher was paired up with parents at T1 and T2.

Data collection for T1 lasted approximately 30 min per participant and was organized after the information and consent meeting. The demographic information collected at baseline included participants’ age, nationality, mental health diagnosis (if applicable), postcode (as a proxy for socioeconomic status), referral source, as well as marital, educational, and employment status alongside their children's age, gender, residence, and contact status. Symptoms of psychological distress at the beginning of the program were captured via the Brief Symptom Inventory‐18 (BSI‐18; Derogatis, 2001), an 18‐item self‐report measure. The BSI‐18 consists of three subscales that assess individual symptom constellations (Depression, Anxiety, and Somatization) and one overall subscale that captures the intensity of global psychological distress (Global Severity Index) during the last 2 weeks. Items in this scale can be scored from “Not at all = 0” to “Extremely = 4″. Raw scores were converted to T‐scores based on gender‐specific normative data from a non‐clinical population. The reliability and validity of this questionnaire have been demonstrated in several studies for both community and clinical populations (Meijer et al., 2011; Recklitis et al., 2006). For the current sample, reliability at T1 was α = .90 for the overall scale and α = .80, .83, and .74 for subscales, respectively. Participants’ attendance and involvement with child protective services were recorded by group facilitators and shared with the research team post‐intervention.

Data collection for T2 was scheduled towards the end of the program, between weeks 12 and 14, and each session lasted approximately 45 min. The interviews were conducted after completing the questionnaires and lasted between 15 and 35 min. A semi‐structured interview guide was developed prior to interviews in line with best practice guidelines (Mason, 2004). The guide covered five broad areas of interest: context before the intervention, change for parent and baby following the intervention, parent‐baby relationship, the experience of attending MB and future plans (see Appendix 1). The guide provided a focused structure for the interviews; however, researchers were encouraged to explore avenues of discussion raised by participants, as Kallio et al. (2016) recommended. All interviews were audio‐recorded for research purposes.

### 2.5 Data analysis

Interviews were transcribed verbatim and anonymized. All researchers were familiar with the transcripts, but analysis of the interviews was primarily conducted by an independent MSc researcher (CD) and supervised by a senior researcher (AMacB) who were not involved in the data collection, mitigating any risk of bias. A thematic analysis (TA) was employed to systematically identify, analyze, organize and report shared themes and patterns within the dataset (Braun & Clarke, 2006). TA was selected as a method for exploring participants’ thoughts, feelings and behaviors, allowing for similarities and differences in experience to be uncovered without being constrained to a theoretical model (Braun & Clarke, 2006; Joffe, 2012; Nowell et al., 2017). Given the exploratory nature of the research, it was deemed that a flexible approach, as TA is, would be the most beneficial for understanding the participant experience of MB.

This study followed the TA protocol outlined in Braun & Clarke (2006) and Nowell et al. (2017). Following transcription, the analysis involved an iterative process of familiarizing with the data (actively reading through interviews while making initial notes). Transcripts were imported to NVivo software (Version 13, 2020) and coded line‐by‐line, paying attention to descriptive, linguistic and conceptual elements (Braun & Clarke, 2006). A second and third‐order analysis of coding was then completed to ensure the rigor of coding (Fereday & Muir‐Cochrane, 2006). Thereafter, codes were grouped to form themes and sub‐themes. Following Braun & Clarke (2006) suggestion, themes were identified based on both prevalence and “keyness” (i.e., the ability of the theme to capture what is important considering the research questions), given that higher prevalence alone does not necessarily equate to higher importance. To establish trust and confidence in the findings of this research, interrater reliability was employed in the form of triangulation, whereby one‐third of transcripts were also coded by another member of the research team (AMacB). Final codes and themes were discussed with all the research team members to reach a consensus and ensure the validity of the analysis.

## 3 RESULTS

### 3.1 Sample characteristics

**FIGURE 1:**
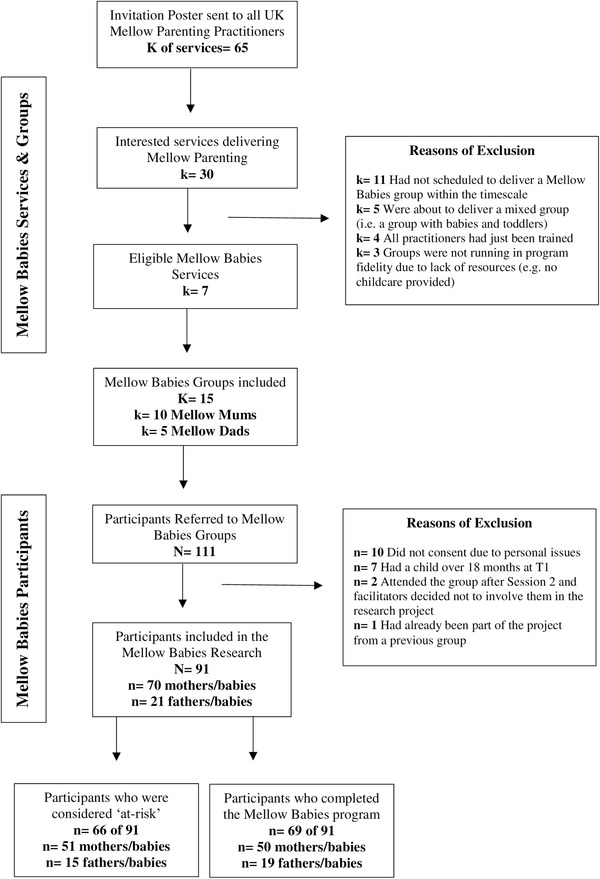
Mellow Babies research project: Services, groups and participants flow chart

As illustrated in Figure 1, of the 111 parent‐infant dyads recruited in the participating MB groups, 91 consented to take part in the research project (70 mother‐infant and 21 father‐infant dyads). Based on the psychosocial criteria set, 66 (73%) of our baseline sample (91) were considered at‐risk, of which 51 out of 66 (72%) were mothers. Of the project participants, 22 dyads did not complete the program giving an attrition rate of 24% (0%–44% range amongst groups). Of non‐completers, 9% (two) were fathers. Of the 69 parent‐infant dyads who completed the MB group, 68 parents were interviewed at T2 (49 mothers and 19 fathers; one mother was not available at T2 data collection). Those who completed the program attended an average of 80% of the 14 MB sessions. No differences were observed in the number of sessions attended, and the intervention effects assessed quantitatively based on risk status and gender (Raouna et al., 2021). No differences were found in the demographic characteristics of the participants who completed the MB program and those who did not. Participants who did not complete the program reported higher parenting confidence at T1 compared to the participants who completed the program (Raouna et al., 2021). Demographic information of the participants who were interviewed is provided in Table 1. Comparison of the demographic characteristics between mothers and fathers using Pearson's chi‐square tests indicated that the only significant difference was that more fathers (63%) than mothers (10%) reported that their child was not living with them at the beginning of the program [χ^2^ (1) = 19.52, *p* < .001].

**TABLE 1:** Baseline demographic information for parents who were interviewed (*n* = 68)

Parent characteristics	Mothers (*n* = 48^e^)	Fathers (*n* = 19)
Mean age in years (SD)	25.69 (5.94)	28.68 (8.85)
Nationality (*n* [%])		
British	42 (87.5)	17 (89.47)
Other^a^	6 (12.5)	2 (10.53)
Education (*n* [%])		
Still at school	1 (2.08)	1 (5.26)
Did not finish school	5 (10.42)	2 (10.53)
Secondary school	17 (35.42)	10 (52.63)
College	18 (37.5)	5 (26.32)
Further education	7 (14.58)	1 (5.26)
Employment (*n* [%])^b^		
Full‐time employment	7 (14.89)	3 (15.79)
Part‐time employment	5 (10.64)	0 (0)
Unemployed ‐ with benefits	24 (51.06)	13 (68.42)
Unemployed ‐ no benefits	11 (23.41)	3 (15.79)
Relationship status (*n* [%])		
Single	24 (50)	5 (26.32)
Married	9 (18.75)	4 (21.05)
Co‐habiting	11 (22.92)	6 (31.58)
In a relationship, not co‐habiting	4 (8.33)	4 (21.05)
Mental health diagnosis (*n* [%])^c^		
None	21 (46.66)	12 (63.16)
Depression	9 (20)	2 (10.53)
Anxiety disorder	7 (15.56)	0 (0)
Depression & anxiety	7 (15.56)	3 (15.79)
Other	1 (2.22)	2 (10.53)
Current psychological distress (above clinical cut‐off; *n* [%])^d^		
Depressive symptoms	16 (32.65)	7 (36.84)
Anxiety symptoms	30 (61.22)	12 (63.16)
Somatization symptoms	10 (20.41)	4 (21.05)
Global severity index (GSI)	16 (32.65)	7 (36.84)

Child in group characteristics	**Mothers (*n* = 48** ^e^)	**Fathers (*n* = 19)**
Mean age in months (SD)	8.70 (7.15)	11 (5)
Gender (*n* [%])		
Female	20 (41.67)	10 (52.63)
Male	28 (58.33)	9 (47.37)
Residential status (*n* [%])		
Lives with parent	43 (89.59)	7 (36.84)
Kinship care	1 (2.08)	7 (36.84)
In foster care	3 (6.25)	3 (15.79)
Supervised contact with the parent	1 (2.08)	2 (10.53)

^a^Other = British‐Asian (*n =* 1), Dominican (*n =* 1), Malaysian (*n =* 1), Moroccan (*n =* 1), Nepalese (*n =* 1), Pakistani (*n =* 1), Zimbabwean *(n =* 2).

^b^ *n* = 47 for mothers, fathers *n* unchanged.

^c^ *n* = 45 for mothers, fathers *n* unchanged, as listed by parents.

^d^ *n* = 49 mothers, fathers *n* unchanged, as reported on the Brief Symptom Inventory‐18; parents were categorized as a clinical case if the T‐score of the subscale was ≥63 and for GSI if at least two of the subscales had a T‐score ≥ 63 (Derogatis, 2001).

^e^ Demographic details absent for *n* = 1 mother and *n* = 1 child in the total sample.

### 3.2 Themes

Three themes and six subthemes were generated from the data, as shown in Table 2.

**TABLE 2:** Themes, subthemes and extended subthemes with the number of parents endorsing each and illustrative quotations

**The parental journey**	**Benefitting baby**	**Moving on from Mellow**
**From hesitance to engagement** Isolation (32) Mental health difficulties (35) Challenges adapting to parental role (20) Impact on socioemotional development of baby (4) Eager to join group (3) Low expectations of group (34) Comparing to other groups (6) Facilitator role (30) Facilitator support (8) Re‐engagement with services (4) People who understand (60) Gender‐specific groups (5) Sharing in a homogeneous group (39) Cultivating community (45) “You can go to groups and sit quiet, and everybody else talks – with Mellow it's different, you get a chance to have your say and your opinion matters. You weren't sitting there feeling silly or embarrassed” [Dad 15] **“Gaining a bit of me back”** Improved mental health (43) Maternal identity (15) Reduced stress (21) and emotional regulation (33) Confidence (54) Social connectedness (58) No reported change (1) “Working on ourselves really helped me moving on with my postnatal depression, in terms of speaking about how I was feeling and thinking, actually it's okay to open up. So in terms of me, even mentally for me moving on, it was a great help” [Mum 36] **“Breaking that cycle”** Life stories (49) Experience of being parented (10) Recognizing abusive relationships (8) Disconnected from content (2) “We looked deeper into our past when we were children, that was difficult for me because there's stuff happened in my childhood that I would never bring to the front of my memory, but I suppose it was good because it's stuff that you don't face and then when you have to, it's really good to realise that you've faced it and you can get over it and you can get on” [Mum 42]	**The parent‐child relationship: Reshaping interactions** Understanding baby (38) Mindful and playful (17) Video feedback (30) Increased love (2) Infant's confidence (5) No change to relationship (6) “I wasn't sure about being around him, I didn't feel I had a connection with him, I didn't feel there was a bond there with him, I wanted to foster him out at the beginning – but now, I just love him to bits” [Mum 11] **“My baby is a lot happier”** Widening social world (10) Social development (35) Mental health (25) Confidence (9) Positively impacting relationships (12) “She was clingy before, she wouldn't leave me alone and now she's started to leave me and will do her own stuff. Before she would cling on to me, she wouldn't move, she wouldn't leave me alone, she wouldn't let anybody else pick her up or that but now she lets everybody pick her up, she'll go and play with other bairns [children]” [Dad 2]	**“I don't want to be on the dole my whole life”** Group extension (15) Repetition (13) Desire to learn (7) Lack of confidence (10) Education (10) Employment (22) Mellow Toddlers (5) Re‐engaging with the community (20) Maintaining friendships (41) “Probably at the start of the group I had never, ever seen a future for me, I never – I found it hard to look into the future, I didn't – it's hard to explain but I just didn't see anything, I was so just to just feeling numb but now I'm much more confident and I see a really good future for me and the girls, so it's just me and my girls. Maybe getting a job in like six months’ time, providing for them, just being safe and happy” [Mum 21]

#### 3.2.1 The parental journey

##### 3.2.1.1 From hesitance to engagement

In line with referral criteria, most parents reported unhappiness due to isolation (*n* = 32*)*, mental health difficulties (*n* = 35), or challenges adapting to the parental role (*n* = 20) before starting the intervention, “I suffer with depression and anxiety and as a mother it did get a bit much for me, I wasn't coping with life and being a mum and just everything” [Mum 21]. These consistent reports of low mood, high‐stress levels and social disconnection were shared equally by mothers and fathers. A few parents also reflected on the negative impact that their wellbeing and functioning had on the relationship with their baby and their socio‐emotional development (*n* = 4), “I was never out, I was always in the house with the wee boy. He wasn't getting to learn anything” [Mum 18].

Pre‐intervention difficulties contributed to negative feelings about attending the group. While a few parents were eager (*n* = 3), the majority reported feeling nervous and having low expectations (*n* = 54). Fathers expressed hesitation about opening up to strangers and feeling pressured by external services to attend (e.g., child protective services), while mothers’ experience was mostly characterized by social anxiety and fear of judgment of their parenting abilities. However, after attending the MB group, most participants commended the relaxed and welcoming environment of the program; a surprising element for some parents who expected to be “reprimanded” or “talked at” (*n* = 13). A small number of parents (*n* = 6) also positively compared their experience at MB with that of other previously attended group‐based parenting groups, “I've been to a couple of groups, and none have made me feel so comfortable, relaxed and as confident as Mellow Dads” [Dad 19].

When parents reflected on the elements that motivated them to complete the MB program, it became apparent that the role of the facilitator was key in creating a “safe” and enjoyable intervention for parents (*n* = 30), “it feels like they're not really leaders, they're just people you go to the group with, it's been a lot more comfortable” [Mum 3]. Facilitators were appraised for their strong interpersonal skills, including being empathetic, open and responsive, which enabled parents to establish a trusting relationship with them and the other group members, making them feel seen and looked after. In a few cases (*n* = 8), parents also referred to gaining an ally even beyond the group, “with my court meetings, I'd went in and there was nine people there for my ex‐partner to support her and I was sitting there myself. The next day [the facilitator] turned up and he was there for me, I had somebody that was beside me and I wasn't alone” [Dad 19]. Specifically for parents involved with social services, MB was perceived as a space that “allowed” them to parent their child, “I didn't feel like I was being watched or judged. I could just be mum to her” [Mum 33]. For some parents (*n* = 4), this positive relationship established with the facilitators created a vehicle to re‐engage with services from which they had previously felt marginalized, creating a positive snowball effect of improved relationships with healthcare‐related professionals, “the social worker said he was proud of me. We get on brilliant with him now. It makes things a lot easier now because [we] didn't like when they first came into the house but now he's welcome!” [Dad 13].

The group‐based element of MB was described as valuable and enjoyable, however, parents recognized that this was mainly because the group was comprised of “people who understand” [Dad 7] (*n* = 60). Gender‐specific groups were noted by dads to be an important aspect of the intervention that encouraged their involvement and enabled them to feel “heard” (*n* = 5). Parental reports (*n* = 39) indicated that the act of sharing experiences within a homogeneous group elicited a realization that they were not alone and reduced emotional isolation, “you're realizing that it's not just you that's been through bad stuff and sharing that with somebody, how you felt ‐ is really comforting” [Mum 18]. Discussion of challenging experiences within the context of the group setting was also seen as an enabler to cultivate a sense of community and belonging that was valued by the majority of parents (*n* = 45), “we've become more of a family now” [Mum 34].

##### 3.2.1.2 “Gaining a bit of me back”

Both mothers and fathers experienced an improvement in their mental health following attendance at MB (*n* = 43), including positive changes in their mood, anxiety and self‐esteem; one to such an extent that they were advised to discontinue their anti‐depressant medication. Maternal reflections (*n* = 15) highlighted the role of MB in enabling them to reconnect with neglected aspects of their identity beyond motherhood and mental health challenges, “I've gained a bit of me back. I thought ‐ “That's it, I don't matter anymore”. But you can only last so long like that and you can feel lonely, sad, depressed. I feel a hell of a lot better now” [Mum 20]. For both mothers and fathers (*n* = 21), perceived improvements in mental health led to a reduction in parenting‐induced stress and enabled them to access more adaptive coping skills and emotion regulation strategies (*n* = 33), “how I'm handling myself whenever situations come up, even [with] the two older ones, I've been more relaxed in my approach with them and their behavior” [Dad 1].

As a consequence, parents reported feeling more confident both in their daily life activities, “I didn't like going to big public spaces, I just felt everybody was talking about me and looking at me” [Dad 16] and in their parenting skills following MB attendance, “I used to ask my mum for a lot of help ‐ I felt I wasn't able to do it myself whereas now I feel I can” [Mum 46]. This confidence boost contributed to reduced isolation via increased social connectedness and ability to engage with the community for both mothers and fathers (*n* = 58), “I'm more sociable, I have more friends, I can talk to people. I'm back out in the community again rather than being so isolated and having nobody” [Mum 14]. However, it should also be noted that one mother [Mum 2] reported experiencing no change in her wellbeing. This appeared to relate to the feeling that the group was not relevant to her needs and therefore, feeling disconnected from other group participants.

##### 3.2.1.3 “Breaking that cycle”

Parental reflections indicated that open yet moderated conversations around sensitive topics within the group provided them with a new lens from which they viewed personal adversity and relationships. Both mothers and fathers (*n* = 49) mentioned the “Life Stories” session as being an emotionally challenging yet vital component of the intervention that held therapeutic value and enabled psychological processing of difficult memories and emotions, “it's really good to realize that you've faced it and you can get over it” [Mum 42]. “Life Stories” provided parents with the opportunity to coherently formulate and share their experiences, allowing them to make connections with their current functioning, “looking back and to where we were today because of what's happened” [Mum 19]. For some parents, this also initiated an introspection around their experiences of being parented, identifying parenting practices they would like to maintain or avoid moving forward (*n* = 10), “if there was something like this for my mum, I wouldn't have ended up in the same position she ended up, being here has broke that cycle for my daughter” [Mum 17]. The nature of these discussions enabled parents to increase their understanding of complex interpersonal relationships and, in some cases, to recognize that they had been a victim (*n* = 6) or perpetrator (*n* = 2) of abuse in romantic relationships, “I was so blinded by my ex I didn't even realize what he was doing was wrong” [Mum 22]. Both victim and perpetrator reflections indicated that understanding the patterns of abusive behavior made them feel more confident that in the future they would be able to avoid similar relationships, “if the same thing was to happen again, I would know the cues and I would know how to spot it” [Mum 37]; “it's something I'll be able to combat now” [Dad 1].

Of note, two parents reported that they did not enjoy discussions around interpersonal adversity, criticizing the content and format of the group as being largely focused on past experiences and expressing a desire for a more uplifting and future‐focused element to be weaved into the intervention, “it's kind of been a negative experience because we're just permanently going over the crap that we've been through in life” [Mum 2]. Such reflections pointed to the importance of group members having a shared background and indicated that MB may be more beneficial for parents who are at the “same stage” of processing their experiences “if you're somebody that hasn't worked out your issues, hasn't put the past to bed, I think this is a good opportunity to come to share your stories. But I think that when you've dealt with issues and with past negative experiences and you want to look forward the group picks up the stuff that you want to forget about” [Mum 2].

#### 3.2.2 BENEFIT TO BABY

##### 3.2.2.1 The parent‐child relationship: Reshaping interactions

Maternal reflections (*n* = 38) indicated that after attending the group they had an increased understanding of their child's needs, mental states and developmental stages, which was perceived as leading to more positive interactions with them, “exercises like that made me put myself into the bairn's [child's] shoes and I understood what I would be able to do and wouldn't be able to make her more comfortable or uncomfortable” [Mum 17]. Parents shared that the child‐centered psycho‐education included in the program equipped them with the knowledge and skills to enhance the quality of their interactions, becoming more mindful and playful (*n* = 17). Specifically, the majority of mothers (*n* = 30) highlighted that by being supported through the strength‐based video feedback exercises and the weekly guided interaction activities, they experienced benefits on multiple levels, including increased confidence in their parenting capacity and stronger connection and attunement with their child, “I understand her cues more [now]. I couldn't really pick up a lot but now with the help of everybody, I've been able to pick up the cues that she's tired, she's hungry, she wants a cuddle” [Mum 31]. This also empowered parents to relate to their infants in a more engaging and developmentally appropriate manner, “before I would say what am I meant to be doing with him? What sort of things am I meant to be playing with him? Now, I'll chase him on his bike, paint or draw” [Mum 18].

A small number of mothers (*n* = 2) discussed feeling increased love towards their infant post‐intervention, “I love [my son] more than anything now whereas to think back two years, I didn't ken [know] if I loved him” [Mum 18]. Parents also reflected on the reciprocal nature of these benefits, highlighting the bi‐directional impact of parent‐child relationships, “it seems like a lot more love and attachment, both ways rather than just feeling like he just needs me it's like he actually likes me!” [Mum 27]. For example, a few parents (*n* = 5) indicated that their infants increased their confidence in exploring their surroundings and in trusting them following MB, “I go up to my pal's house from the group and she'll just walk away and leave me, whereas before she would cling on to me, she wouldn't move, she wouldn't leave me alone” [Dad 2]; “I can see the trust he has in me, I can see that he understands that if I go to the toilet he knows I'm going to come back” [Dad 19]. A small number of parents (*n* = 6) reported no change in the relationship with their child, however, these parents also reported having a strong relationship with their child prior to starting the intervention.

##### 3.2.2.2 "My baby is a lot happier”

Several parents witnessed a positive widening of their infant's social world post‐intervention, with some parents (*n* = 10) reporting that their baby had never played with other children before attending MB. Parents, in the vast majority mothers, highlighted that the attendance of their children at the childcare group that runs parallel to the MB group had benefitted their infant's social development (*n* = 35), mental health (*n* = 25) and confidence (*n* = 9), “my baby is very sociable now whereas beforehand he would have been strange with people” [Mum 25]; “he learned how to smile” [Mum 34]. The opportunity for infants to socialize within the childcare group was also noted to positively impact upon the relationships of the babies beyond the MB group with siblings and other family members (*n* = 12), “I've got a three‐year‐old sister and he wouldn't bond with her but after going to this group he bonds with her so much and it's amazing to see that bond there, playing with her” [Mum 13].

#### 3.2.3 Moving on from Mellow

##### 3.2.3.1 "I don't want to be on the dole all my life”

When considering their plans and goals following MB, several parents expressed a desire for the group to be extended (*n* = 15) or to repeat the group entirely (*n* = 13), “I would give anything to come back to another Mellow Dads group” [Dad 12]. There were both maternal and paternal reports wishing the group would “go on forever”, with some parents indicating that their desire to repeat the group was embedded in the opportunity to learn more (*n* = 3), “extra learning, that's what I'll miss the most” [Dad 11], or to make sure they had not missed any important elements (*n* = 4), “when they start the Mellow Dads up again I'll probably ask to attend again, just in case there's anything I've maybe missed” [Dad 12]. A few parents expressed apprehension and a lack of confidence regarding their future (*n* = 10), expressing doubts about the lasting effects of MB on their lives and feeling insecure without the safe haven that the group offered to them, “I've got a bit more confidence but it's not enough to keep me going, as soon as this group's finished I'll probably be going back in again. I don't know what I'll do without this group, I don't want it to finish” [Mum 10].

On the other hand, some parents shared that the group provided them with motivation and hope for their futures, “I don't want to be on the dole all my life and this group has said “you can get a job and make your daughter proud”, no‐one's ever said that. No‐one's ever pushed me towards doing it” [Mum 13]. Approximately half of the parents expressed a desire to continue developing autonomy through the acquisition of new skills either by moving into education (*n* = 10) or seeking employment (*n* = 22). Parents reported feeling empowered by the support and guidance received from other group members and specifically group facilitators to explore employment and education opportunities available to them post‐intervention, “it's really, really helped me, I mean if you'd said to me a year ago “In a year's time you're going to be making an appointment with the Job Centre, I'd have went “No, I'm not!”’ [Dad 16]; “I've been talking to [the facilitator] and they're going to put me forward to do a child care course” [Mum 42]; “it's sort of showed me what I want to do. I'm going to try and get into uni” [Dad 18].

Acknowledging their need for continued personal and parenting support, some parents also planned to move onto Mellow Toddlers (*n* = 5) within the same community setting, while many parents (*n* = 20) appeared to have the confidence to re‐engage with the wider community by attending other support and parenting groups, “I am planning on taking the kids to other toddler groups in the area” [Mum 26]. Lastly, the majority of mothers and fathers (*n* = 41) expressed a desire to maintain the friendships acquired throughout the group; indicating a sustained increase in proactive social connectedness, “I'm going to keep in touch with most of them here. I hope to try to organize an actual dads’ group myself ‐ go to the soft play or go swimming” [Dad 11].

## 4 DISCUSSION

This is the first UK‐wide study to provide qualitative evidence of the parental experience of attending the MB program for both mothers and fathers, offering insights into aspects of the intervention that may facilitate change for at‐risk families. Parents who completed the program shared a positive experience for themselves, their relationship with their children, and the socio‐emotional development of their babies, with mixed thoughts shared about their post‐intervention plans. This largely aligns with quantitative findings from this project, demonstrating that completion of the MB group offers significant improvements in anxiety levels and overall wellbeing, parenting confidence and perceived closeness of the parent‐child relationship (Raouna et al., 2021). Parents identified multiple, interlinked components of the intervention as enabling positive change and engagement, including: (i) the group‐based nature of the intervention and specifically its supportive environment and homogeneity in terms of psychosocial and gender characteristics, (ii) the role and interpersonal skills of facilitators, and (iii) the multi‐dimensional focus of the intervention, including opportunities for personal reflection, guided positive interaction with their babies and provision of a parallel childcare group. Overall, the findings of this study highlighted three main processes through which MB may benefit at‐risk families: (i) the sense of community cultivated within the group, (ii) the process of formulating and re‐conceptualizing one's own difficulties, and (iii) the opportunity of reshaping interpersonal interactions.

### 4.1 Cultivating community within the group 

Consistent with the existing literature, parents valued the social interaction provided by the group‐based nature of MB and identified the acquisition of friendships as an important aspect of the intervention (Butler et al., 2020; Ruane et al., 2019). Cultivating a sense of community was enabled through the discussion of shared experiences in which the inclusive role and interpersonal skills of facilitators were perceived as key to creating a supportive environment within the group. Parents conveyed that the opportunity to discuss difficult experiences with others who “understand” removed feelings of stigmatization and guilt that have been previously reported to function as barriers for at‐risk parents (Webb et al., 2021). Additionally, gender‐specific groups appear to have been especially important for fathers, which is in line with findings suggesting that mixed‐gender groups can feel unwelcoming and intimidating to partners (Smokowski et al., 2018; Panter‐Brick et al., 2014). Fathers reported feeling “seen and heard” within the MB group, disrupting previously reported narratives of fathers feeling like the “invisible parent” by healthcare professionals and services during the perinatal period (Lever et al., 2018).

Overall, perceived reductions in social and emotional isolation held a significant value to the parents completing the MB program, mirroring a vital intervention outcome when engaging parents living within the context of multiple psychosocial adversities (most participants at the beginning of the program lived in highly deprived areas, and/or experienced a mental health issue, were unemployed and single parents). This observation supports the recommendations of employing a balanced approach when working with at‐risk families, offering a space to address underlying psychosocial issues as well as to promote sensitive parenting skills and parent‐infant attunement (Butler et al., 2020; Whittaker & Cowley, 2012). Furthermore, although our findings validate the additional benefits associated with the provision of targeted group‐based interventions delivered within community settings, they also highlight the decisive elements of group members’ synchrony and perceptions of one's situation and difficulties in opening up to the potential benefits of the intervention. Consequently, to maximize intervention effectiveness and minimize any potential harms, it is crucial to have careful assessment procedures to determine whether a parent would benefit more from a 1:1 or a group‐based intervention and provide ongoing opportunities for facilitators to nurture their interpersonal skills (e.g., through reflective consultation).

### 4.2 Re‐conceptualizing one's difficulties 

Parents consistently indicated that the “Life Stories” session, a session strategically placed mid‐way through the program, was a powerful intervention component that may drive change. The opportunity to formulate and reclaim the narrative of one's own life experiences after establishing a trusting relationship within the group provided parents with a safe space to process and re‐conceptualize their difficulties. For some parents, this also functioned as a turning point, leading to an emotional shift from which they felt they could “move forward” (Johnstone, 2018; Redhead et al., 2015). Specifically, parents indicated that the reflective elements of MB allowed them to become aware of past mistakes and maladaptive behaviors both of themselves, their parents, and their (past) romantic partners. This finding aligns with existing evidence suggesting that the provision of reflective opportunities may actively contribute to the prevention of the intergenerational transmission of psychosocial adversity (Schoon & Melis, 2019). However, it should be recognized that sustainable change depends upon a complex interplay between capabilities, motivations, and contextual factors (Platt & Riches, 2016), in which desire alone is insufficient (Byng‐Hall, 2008). Parents indicated that the value of MB did not lie in “prescribing” change for them but in making them realize that circumstances can change and empowering them to generate positive changes for themselves.

As it is not possible to quantify the value of a reflective space using standardized measures, this study has provided valuable insight into what parents consider instrumental in having a positive impact as part of their MB group experience. Nevertheless, it should be noted that a few parents expressed a desire that the content would have a greater emphasis on looking towards the future and “moving on” from difficult experiences. It is, therefore, important to acknowledge that although MB targets parents experiencing psychosocial difficulties, within the current sample, there was still variation in the level of higher education attained, employment status, severity of mental health issues experienced and child‐rearing circumstances within the context of lone parenthood and varying custody arrangements. Such factors could have mediated the differences observed in the perception, engagement, and experience of the intervention, influenced by elements such as attendance motivators (e.g., self‐referred vs. court‐referred) and competing demands (Mytton et al., 2014; Whittaker & Cowley, 2012). Future research may wish to specifically focus on the reflective component of the intervention and explore whether its effectiveness may be moderated by different psychosocial indicators, as our finding is consistent with the wider MP literature, and thus, research is converging to uncover what may be a core ingredient of the intervention's effectiveness for at‐risk families (Birtwell et al., 2015; Buston et al., 2019; Puckering et al., 2010).

### 4.3 Reshaping interpersonal interactions

The development of parents’ connection and communication with their infant was another key change perceived as a consequence of attending the MB group. Parents often attributed these changes to their increased knowledge about child developmental stages and mental states as well as the guided positive interaction activities. Gaining confidence and learning how to play with their child was another important component mentioned by parents; of which the value of nurturing child development and the parent‐child relationship cannot be overlooked (Ginsburg, 2007). Within the current analysis, participants suggested that their parenting confidence increased via the encouragement of trying new activities with their babies and witnessing the reciprocal benefits of being more in‐tune with them. Additionally, some parents reported noticing positive socio‐emotional changes in their young children post‐intervention, both because of attending the parallel childcare group and of their own improved wellbeing and mindfulness. These observations emphasize the dynamic and transactional nature of family interactions (Schermerhorn et al., 2008), reinforcing the need to provide multi‐dimensional interventions.

Interestingly, when fathers were prompted to reflect on their experiences within the group, these were weighted toward their own experience of attendance in contrast to maternal reflections that were mostly focused on the shared impact of attendance on themselves and their infant. This difference could be attributed to the fact that the majority of fathers who completed the group did not have full custody of their child when the group started (which was also the only statistically significant difference in the demographic characteristics between mothers and fathers). This may have limited their opportunity to witness changes in their child's socio‐emotional development across different contexts. Nevertheless, for the parents participating with their child in someone else's custody (25%), reflections indicated that MB provided them with a trusted and non‐judgmental environment to parent, positively impacting their experience of the intervention and their relationship with their infant. In fact, more than half of the parents involved with child protection services at the beginning of the group experienced a de‐escalation of their case during or post‐intervention, indicating that statutory sector services, primarily the ones who also referred parents to MB groups, identified signs of improvement in participants’ parenting skills and relationship quality with their children (Raouna et al., 2021). Aside from this, there were no gender differences in the experience of the intervention, which is consistent with findings suggesting that mothers and fathers can be benefited equally from a gender‐tailored group intervention (Raouna et al., 2021). Nevertheless, it should be noted that mothers were overrepresented in the sample and further research is required to identify if there are specific mechanisms that support the involvement of fathers in early intervention programs.

The positive impact of MB in interpersonal relationships extended upon the parent‐child relationship and permeated the wider family context. Parents reflected on improved relationships within the family home as a result of attending MB, including with their own parents and other children. There were also parental reports of adopting a calmer approach when engaging with schools and social services. In some cases, the impact of increased parental confidence also expanded in other aspects of their lives, exemplified by an increased desire to engage with further education, employment, or sustain acquired social connections. In this way, MB appeared to have an attachment function, providing parents with a secure base from which they felt capable of further exploring their world and the opportunities available (Baumeister &, Leary, 1995; Page, 2011).

Considering this function, it is also possible that parental attachment style may have contributed to the pre‐and post‐intervention experiences and feelings expressed by parents. Research suggests that rates of insecure attachment styles are higher amongst at‐risk individuals (Thomson & Jaque, 2017), who may display higher levels of anxiety and be more fearful of new experiences (Goleman, 2006; Markin & Marmarosh, 2010). In this line, our analysis found an unintended consequence of MB participation, with a few parents sharing the desire for the group to renew on a supportive basis; expressing hesitance and low levels of confidence about their future without the sense of stability that the group offered. Although a group extension would run counter to the evidence‐base highlighting an excess of 16 sessions as reducing intervention effectiveness (Bakermans‐Kranenburg et al., 2003), this finding may be an indication of the different levels of support at‐risk parents may need based on the severity of their psychosocial adversity and attachment style. Future research may wish to assess parental attachment style and nuances of psychosocial adversity levels to investigate this theoretical standing, gaining a better understanding of how components such as developing a secure parental attachment can be cultivated within early interventions to facilitate parental autonomy post‐intervention (Duggan et al., 2009).

### 4.4 Limitations and recommendations for future research

While we have uncovered aspects of MB that can encourage engagement, we have been limited in our capacity to understand what disengages parents, given that interviews were conducted only with program completers. Future research should strive to gather information from non‐completers as well to gain a better understanding of the experiences of all participants referred to MB, informing future practice more accurately. The transferability of our findings are also limited to the British‐white population and future research should attempt to replicate findings with a more ethnically diverse sample. Additionally, although the volume of interviews in this project is rare in qualitative research, we acknowledge that the richness and depth of the information shared by parents during the interviews may have been influenced by various factors, including researcher‐participant rapport and/or some interview questions being relatively closed (Boddy, 2016; Gudkova, 2018). Further, while coding was triangulated and the validation of codes and themes was discussed within the research team, the reliability of the analysis could have been stronger had all transcripts been double‐coded and compared during the process of theme development. Nonetheless, we are confident that the findings presented in this study are robust given the consistency of parental reports despite the variance in geographical locations.

Reported improvements occurred within a real‐world delivery of the program, indicating ecological validity. Nevertheless, future research should also strive to triangulate parental‐reported changes against facilitator reports to gain a more holistic understanding of the intervention impact and, if possible, replicate findings via a randomized control trial to further enhance the evidence base of MB. Promisingly, many parents reported that they planned to maintain acquired friendships post‐intervention and that positive parenting practices had been transferred from the MB group to their homes. However, longitudinal research is required to investigate if and how gains are maintained. Lastly, given that MP, adapting to the circumstances, transitioned their program delivery to an online format (www.mellowparenting.org/online‐programmes/), it would be important to investigate whether offline key processes identified in this project (e.g., cultivating a sense of community) are transferrable and experienced by parents participating in digital intervention formats. Understanding the effectiveness and unique characteristics of digital interventions would potentially offer the opportunity to cost‐effectively reach parents residing in locations where face‐to‐face groups may not be available.

## 5 CONCLUSION

The perinatal period offers a critical window of opportunity to identify and address psychosocial concerns via evidence‐based early intervention and prevention programs (Parkes et al., 2016). If these concerns are left unaddressed, they not only pose significant and lasting implications for parents’ mental health, parenting capacity, and quality of parent‐infant interactions but also for their infant's health and development that may have intergenerational impacts. Current findings, collating the experiences of 68 parents who completed the MB program, highlight MB as an intervention that is both accessible and acceptable to at‐risk families, a population that has typically been overlooked by group‐based interventions (Smokowski et al., 2018; Utting et al., 2007). This study, guided by the voices of those with lived experience, uncovered additional elements of MB that drive positive change in the lives of at‐risk families and can function as enablers in re‐engaging them with services and the wider community. Our findings provide valuable directions for future perinatal and infant mental health policy and practice, as well as highlight areas for further research to understand the effectiveness of group‐based early intervention programs for at‐risk families.

## CONFLICT OF INTEREST

Aigli Raouna, Ruaridh Malcolm, and Raquib Ibrahim were paid employees of Mellow Parenting during research conduction. AMacB is a member of the Steering group of the NHS Scotland Managed Clinical Network for Perinatal Mental Health and has received funding from MRC and CSO for maternal and infant health related projects. There are no patents, products in development or marketed products in association with this research to declare.

## Supporting information



Supplementary information

## Data Availability

The data that support the findings of this study are available from the corresponding author upon reasonable request.
