# Supplementary material for: “There's more love between us”: The parental experience of attending Mellow Babies, a targeted, early intervention program for parents and their babies
Source: Infant Ment Health J. 2022 Dec 15;44(1):100–16. doi: 10.1002/imhj.22029 (PMC10107458; doi:10.1002/imhj.22029)
Supplement: Supplementary file 1 — Supplementary information [file IMHJ-44-100-s001.docx]

**Appendix 1**

***Interview Schedule***

These questions should all be asked during the course of the interview. However, the order is not vital and deeper exploration of responses is advised where you feel it is of relevance to the interview. Participants should be encouraged to talk freely and openly in order to accurately share their experiences of the group.

1. Can you remember how things were for you before the group started?
2. Has anything changed for you since coming to Mellow?
3. a. How did you feel about starting the group?
4. Was the group what you expected?
5. Do you feel you have gained anything from the group?

-If so, what?

-If not, why?

1. a. How would you describe your relationship with your baby?

b. How is it different compared to before the group?

1. What did you enjoy most about coming to the group?
2. Did you find any part of the group difficult; If so what?
3. Would you change anything? How could we make it better?
4. a. Would you recommend the group to other mums/dads in similar situations?

b. How would you describe Mellow to someone in a similar situation?

1. Now that you have completed the group, what are you plans for the future?
